# Repeated courses of sequential venetoclax and donor lymphocyte infusions in a patient with relapsed high-risk myelodysplasia following allogeneic stem cell transplantation: a case report

**DOI:** 10.3389/fimmu.2026.1839984

**Published:** 2026-07-02

**Authors:** Federica Gigli, Valentina Fabiola Sangiorgio, Valentina Tabanelli, Giuliana Gregato, Francesco Bertolini, Alessio Maria Edoardo Maraglino, Simona Sammassimo, Rocco Pastano, Enrico Derenzini, Corrado Tarella

**Affiliations:** 1 Oncohematology Division, IEO, European Institute of Oncology IRCCS, Milan, Italy; 2 Haematopathology Division, IEO, European Institute of Oncology IRCCS, Milan, Italy; 3 Laboratory of Haemato-Oncology, IEO, European Institute of Oncology IRCCS, Milan, Italy; 4 Department of Health Sciences, University of Milan, Milan, Italy

**Keywords:** allogeneic hematopoietic stem cell transplantation, donor lymphocyte infusion, post-alloHSCT disease recurrence, therapy-related myelodysplasia, venetoclax

## Abstract

**Background:**

Allogeneic hematopoietic stem cell transplantation (allo-HSCT) represents the only potentially curative therapy in patients with high-risk myelodysplastic syndrome (MDS) and acute myeloid leukemia (AML). However, a considerable proportion of patients experience post-transplant relapse, a condition associated with relevant morbidity and an overall dismal prognosis. In this context, the administration of donor lymphocyte infusion (DLI) has been historically used to exploit the graft-*versus*-leukemia (GvL) effect of allo-HSCT. More recently, hypomethylating agents (HMAs) and venetoclax (VEN), a combination used in first-line/relapsed AML, has also been applied, alone or in association with DLI, for post-transplant relapses, with some encouraging results. Despite several reports showing the feasibility and efficacy of the HMA-VEN combination, the long-term outcome is still poor and the management of AML relapse following allo-HSCT remains unsatisfactory. Thus far, the ideal treatment has not been defined.

**Case description:**

We report here the successful treatment of a patient with high-risk MDS and relapse onset following allo-HSCT. The patient was started on salvage treatment with azacitidine (AZA) and low-dose VEN. Surprisingly, bone marrow cellularity was restored, with rapid improvement of the hematological parameters and recovery of full chimerism as well after a single initial course of AZA/VEN. Two additional courses of AZA/VEN supplemented with DLI were delivered, and then the patient was placed on a maintenance treatment with the sequence of VEN single agent tightly followed by DLI. Six courses of combined VEN/DLI were delivered at 3-month intervals. This innovative strategy proved to be effective in terms of disease control, absence of infectious complications, and quality of life. The patient is presently alive and well in continuous complete remission (CR) 24 months since disease recurrence. The details of his recent clinical history are reported here.

**Conclusion:**

Recurrent courses of VEN in combination with DLI may be exploited for CR maintenance in patients with MDS/AML managed for disease recurrence after allo-HSCT.

## 1 Introduction

Allogeneic hematopoietic stem cell transplantation (allo-HSCT) represents the only potentially curative therapy in patients with high-risk myelodysplastic syndrome (MDS) and acute myeloid leukemia (AML) (1, 2). However, a considerable proportion of patients experience post-transplant relapse, a condition associated with relevant morbidity and an overall dismal prognosis (2–5). In this context, the administration of donor lymphocyte infusion (DLI) has been historically used to exploit the graft-*versus*-leukemia (GvL) effect of allo-HSCT (6–8). More recently, hypomethylating agents (HMAs) and venetoclax (VEN), a combination used in first-line/relapsed AML, has also been applied, alone or in association with DLI, for post-transplant relapses, with some encouraging results (9–16). Despite several reports showing the feasibility and efficacy of the HMA-VEN combination, the long-term outcome is still poor and the management of AML relapse following allo-HSCT remains unsatisfactory, and the ideal treatment has not been defined so far. We report here the successful long-term outcome of a patient with high-risk MDS and relapse following allo-HSCT using a novel approach combining azacitidine (AZA), low-dose VEN, and DLI for re-induction/consolidation followed by prolonged VEN/DLI maintenance therapy.

## 2 Main patient information

The patient in the present report is a white male who had a diagnosis of anaplastic large cell lymphoma (ALCL), *ALK*-negative, at the age of 58 years. He underwent chemotherapy with CHOEP (cyclophosphamide, doxorubicin, vincristine, etoposide, and prednisone) and chemo-mobilization with combined high doses of methotrexate and cytarabine followed by consolidation with autologous bone marrow transplant, obtaining complete remission (CR). After 7 years, while in continuous CR (cCR) of the ALCL, a routine cell blood count (CBC) revealed white blood cells (WBCs) at 2,700/mm^3^ with absolute neutrophil count (ANC) of 1,050/mm^3^. The remaining CBC was unremarkable. Due to suspicion of a secondary myelodysplasia (sMDS), a complete bone marrow examination was performed. Histologic examination of the bone marrow trephine biopsy revealed a hypocellular marrow for the patient’s age (mean cellularity, 20%), with a population of CD34-positive (CD34^+^) blasts accounting for 11% of the cellularity. Flow cytometry (FC) detected a population of CD34^+^ myeloblasts comprising 3.4% of the WBCs. Next-generation sequencing (NGS) on the bone marrow aspirate detected a pathogenic *RUNX1* c.374delC p.(Pro125GlnfsTer8) mutation with variant allele frequency (VAF) of 7.5%. Conventional karyotyping was 46,XY. Molecular analysis revealed a polyclonal status of the T-cell receptor. The overall findings were consistent with a myelodysplastic syndrome with excess blasts type 2 (MDS-EB-2) therapy-related according to the International Consensus Classification 2022 (17).

The patient underwent induction chemotherapy with idarubicin (10 mg/m^2^, days 1 and 3), fludarabine (30 mg/m^2^, days 1–5), and cytarabine (1,000 mg/m^2^, days 1–5), followed by a reinfusion of autologous hematopoietic stem cells (CD34^+^, 10 × 10^6^/kg) spared from the harvesting procedure during the previous treatment for ALCL. Post-induction bone marrow examinations showed CR with no evidence of *RUNX1* mutation. Allo-HSCT represents the only potentially curative therapy for patients with high-risk MDS and leukemiaAML (1, 2). Thus, following a conditioning chemotherapy with treosulfan (1,400 mg/m^2^ die, from day −6 to −4) and fludarabine (30 mg/m^2^, from day −6 to −2), the patient underwent haploidentical allo-HSCT from a non-identical twin brother. Cyclophosphamide was given at 50 mg/kg per day on days +3 and +4, and tacrolimus plus mofetil mycophenolate was started from day +5 forward. Post-transplant bone marrow examinations showed persistent CR with full donor chimerism.

**Figure 1:**
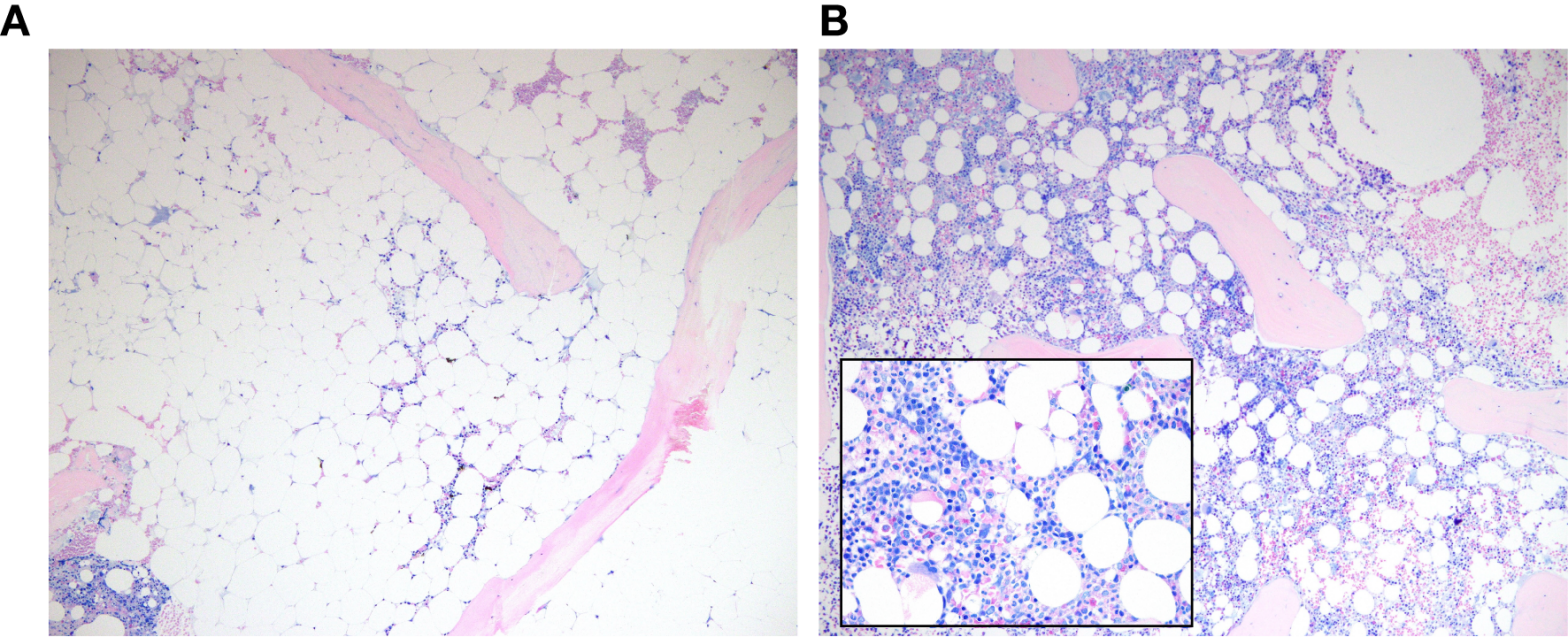
Bone marrow histologic biopsies of the reported case. **(A)** Bone marrow biopsy obtained at the time of post-allogeneic hematopoietic stem cell transplantation (allo-HSCT) relapse showing a severely hypocellular marrow, with abolished trilinear hematopoiesis and interstitial blasts (CD34-positive cell count around 10%, *not shown*). **(B)** Bone marrow biopsy obtained after the first azacitidine and venetoclax course showing recovery of marrow cellularity (overall cellularity of approximately 40%), with maturing trilinear hematopoiesis (CD34-positive cell count <1%; *not shown*). Giemsa stain, ×100 magnification (*inset*, ×400 magnification). Microphotographs were acquired using an Olympus BX53 light microscope equipped with an Olympus DP71 camera.

At 44 months since allo-HSCT, the patient developed a progressive neutropenia (nadir ANC = 930/mm^3^) and anemia (nadir hemoglobin = 11.0 g/dl). A bone marrow evaluation at this time showed a severely hypocellular marrow (cellularity = 5%) with approximately 10% of interstitial CD34^+^ blasts on immunohistochemistry (**Figure 1A**). FC on the bone marrow aspirate revealed a population of CD34^+^ myeloblasts with abnormal phenotype (CD34^+^, CD117^+^, aberrant phenotype, CD45 weak intensity, CD33 weakly expressed, CD13^-^HLA-DR^+^, CD38^+^, and CD123^+^) accounting for 2.6% of the WBCs. NGS detected the known *RUNX1* mutation with a VAF of 5.4%. Chimerism was 95.3% donor.

## 3 Management of post-allo-HSCT disease recurrence

The severe marrow hypocellularity with abnormal blasts coupled with the cytopenias, along with the reappearance of the *RUNX1* mutation and the initial loss of chimerism, was consistent with disease recurrence. Therefore, the patient was started on salvage therapy with AZA (75 mg/m^2^, days 1–7) and low-dose VEN at 20 mg/day for 14 days, along with posaconazole as antifungal prophylaxis (3–9). The VEN dosage was adjusted due to the marked increase in bioavailability upon concomitant administration of posaconazole, a strong CYP3A4 inhibitor (18). AZA/VEN induced a prolonged neutropenia, with 20 days of grade 4 and 5 days of grade 3 neutropenia. However, no infections were reported. Bone marrow examination performed after the first cycle revealed an unexpectedly recovered bone marrow cellularity (cellularity of approximately 40%) with maturing trilinear hematopoiesis and normal CD34^+^ count with minimal (0.1%) persistence of aberrant phenotype (**Figure 1B**). The *RUNX1* mutation was no longer detectable at NGS and chimerism was full donor. In addition, the CBCs improved, with resolution of the pre-treatment cytopenias. The clinical findings were consistent with hematological CR, according to the standard European LeukemiaNet (ELN) criteria. Because of the good response obtained with the first cycle of AZA/VEN, the patient was continued on two additional cycles with the AZA/VEN combination, followed 2 weeks later by DLIs (CD3^+^ at 0.5 × 10^6^/kg at the first infusion and CD3^+^ at 1 × 10^6^/kg CD3^+^ at the second infusion). Neutropenia was observed for a few days following these additional cycles (maximum 4 days of grade 4 neutropenia). Again, no infectious complications occurred.

**Figure 2:**
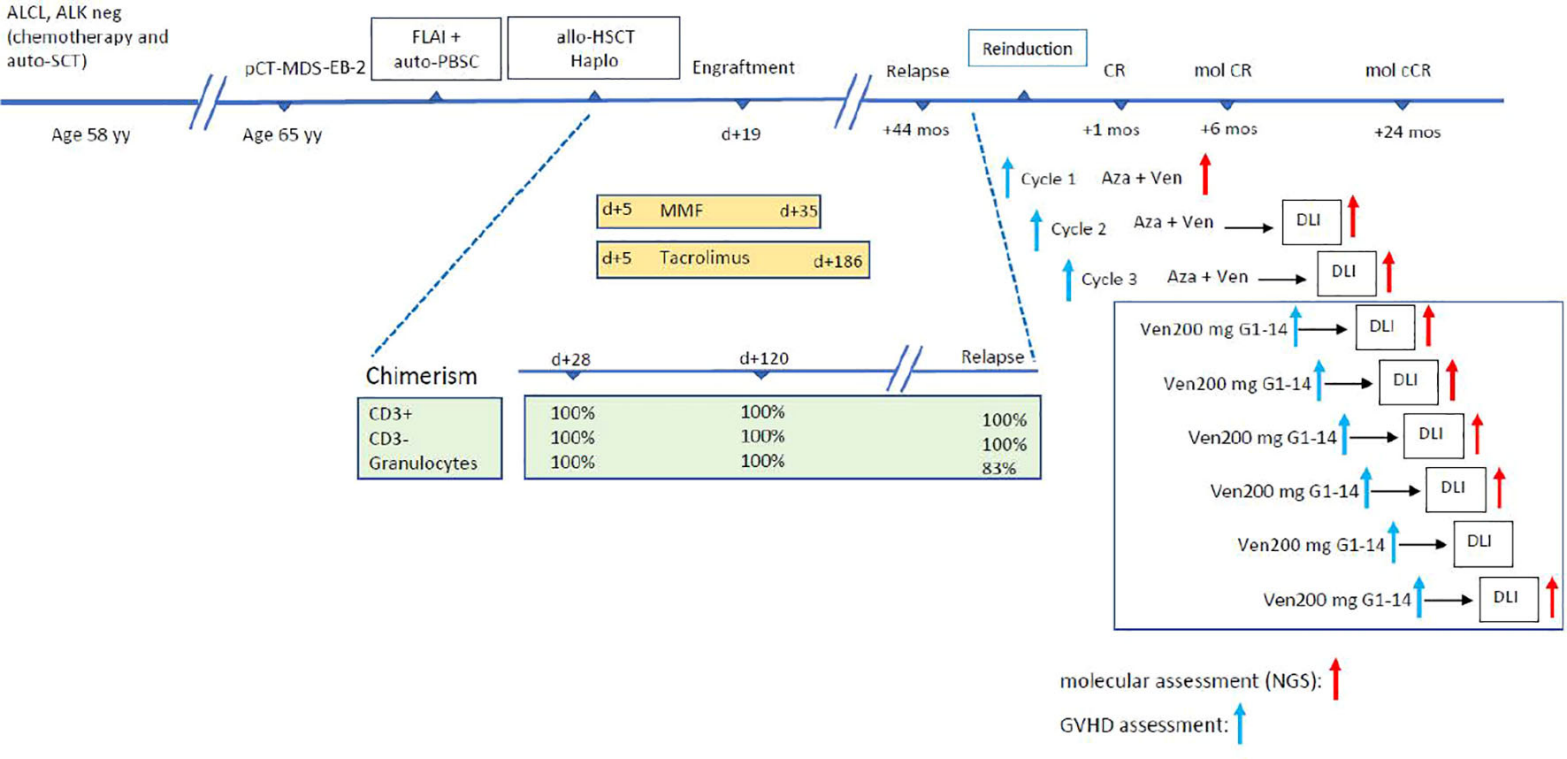
Timeline summarizing the entire clinical history and treatments of the reported clinical case. ALCL, anaplastic large cell lymphoma; auto-SCT/PBSC, autologous peripheral stem cell transplant; pCT-MDS-EB2, post-therapy myelodysplastic syndrome with excess of blasts-2; FLAI, fludarabine, cytarabine, idarubicin; Allo-HSCT, allogeneic hematopoietic stem cell transplantation; MMF, mycophenolate mofetil; Aza, 5-azacitidine; Ven, venetoclax; DLI, donor lymphocyte infusion; d, day; mos, months; yy, years.

At present, the long-term outcomes of patients with MDS/AML recurring after allo-HSCT remain quite poor, and the ideal treatment has not been defined so far (2–5, 16, 19, 20). With the aim of reducing the risk of leukemia recurrence, a maintenance therapy was started with low-dose VEN as a single agent given at 200 mg for 14 days, followed by DLI delivered approximately 14 days after VEN completion. DLI was administered without graft-*versus*-host disease (GVHD) prophylaxis as the patient exhibited no evidence of acute or chronic GVHD following transplant. The low-dose azole prophylaxis was discontinued for two reasons: absence of infectious complications and some signs of drug intolerance. Due to the absence of azole, the dosage of VEN was increased to 200 mg, avoiding the typical full dose of 400 mg/day to mitigate the risk of toxicity in a heavily pretreated patient undergoing additional DLI therapy. A total of six successive courses of sequential VEN/DLI have been delivered, thus far, at 3-month intervals, with the administration of CD3^+^ at 2 × 10^6^/kg for each DLI, as detailed in **Figure 2**. The treatment has been well tolerated, with negligible, transient leukocyte reduction. No signs of GVHD have ever been observed. The patient experienced 2 days of grade 4 neutropenia following the second VEN cycle prior to DLI. The remaining VEN/DLI cycles did not result in neutropenia, with only occasional transient grade 2 neutropenia observed following DLI. No infectious complications have been recorded, with the exception of a few flu-like syndromes. CMV-DNA has been regularly monitored, with no evidence of CMV reactivation having been recorded. At the current follow-up 24 months since achieving CR, the patient is in excellent general condition, with no intervening cytopenias or infections. Chimerism has been persistently full donor, with no evidence of disease on the peripheral blood and bone marrow evaluations. The repeated histologic examination of the bone marrow trephine biopsy also revealed an overall conserved trilinear maturation and a population of CD34^+^ less than 1% of cellularity. Notably, the aberrant CD34^+^ phenotype previously described was no longer detectable since achievement of CR. Molecular NGS studies are persistently negative, with no evidence of the known *RUNX1* mutation. The most recent CBCs revealed WBCs at 4,200/mm^3^ with ANC of 2,400/mm^3^. The remaining values were within normal ranges, with hemoglobin (Hb) at 14.8 g/dl and platelets (Plts) at 271.000/mm^3^.

## 4 Discussion

Disease relapse after allo-HSCT is a complex clinical condition that is extremely difficult to manage. Treatment approaches are quite variable according to the timing and the type of relapse (21–24). In patients with high-risk disease, a so-called prophylactic therapy can be administered to prevent disease recurrence. In this setting, DLI is the treatment of choice because of its ability to boost a GVL effect, restoring disease control in many patients (7, 8, 25). Due to the documented activity, a possible repeated administration of DLI has been proposed (26–28). In cases of molecular relapses and/or to increase donor chimerism, “preemptive” treatments are used. These can include the use of DLI generally in association with systemic treatments such as AZA alone or in association with VEN (8, 14). In cases of overt disease relapse, DLI alone has not been proven to be sufficiently effective, particularly in cases with high tumor burden. In these cases, systemic treatments are first applied to debulk the disease, and DLI has been proposed to maintain response thereafter (29). More recently, VEN has been investigated either alone or as a combinatory drug as maintenance or prophylaxis in high-risk patients following allo-HSCT (30–35).

In our case, the patient had not received any prophylactic therapy after allo-HSCT, and despite having a high-risk MDS, he remained in persistent remission for approximately 44 months. He then developed signs of disease relapse, presenting with severely hypocellular marrow and abnormal blasts, marked neutropenia, reappearance of his previous *RUNX1* mutation, and worsening of chimerism. The clinical situation was at very high risk for several reasons, including disease recurrence after an allo-HSCT procedure, an underlying therapy-related MDS, a previous exposure to intensive chemotherapy with auto-HSCT, and the presence of an adverse *RUNX1* mutation. Taking into consideration all these aspects, an induction with AZA plus VEN at a low dose was decided. Surprisingly, a single course of AZA/VEN was enough to restore a normal marrow cellularity and a full donor chimerism. Two additional AZA/VEN cycles followed by DLI were then delivered as consolidation. Thereafter, a maintenance program combining VEN and DLI was started. To our knowledge, this is the first report of repeated courses of sequential VEN and DLI as maintenance therapy after CR reinduction following allo-HSCT. Differently from normal hematopoietic precursors, neoplastic blasts in MDS and AML heavily rely for their survival on the BCL2 pathway with upregulation of BCL2 and of its downstream effectors (36). Based on this notion, VEN, a highly selective BCL2 inhibitor, has been widely employed for the treatment of MDS and AML. However, VEN as a single agent has shown minimal efficacy in relapsed/refractory (R/R) AML (37). Moreover, in the post-allo-HSCT setting, a successful strategy includes the combination of tumor cytoreduction with GVL boosting by DLI. Based on these premises, the use of DLI in combination with VEN alone or with AZA/VEN has been proposed as a possible treatment option for post-transplant relapses (14–16, 38, 39). Indeed, DLI has been most often employed with the AZA/VEN combination, as described in a recent retrospective study (40). VEN in combination with DLI has been reported in a cohort of 22 patients as first salvage therapy post-relapse, with a limited number of delivered cycles (38). In our case, the combination of VEN/DLI has been introduced as maintenance therapy following achievement of CR with AZA/VEN at a low dose. The VEN/DLI maintenance has been extremely well tolerated in terms of hematological recovery (stabilization of blood counts) and the absence of main clinical complications including infections and GVHD reactivation. Indeed, the excellent tolerability is peculiar and supports the use of VEN/DLI maintenance compared with other DLI combinations, i.e., with chemotherapy or HMAs. Furthermore, it is quite likely that this combined strategy has maintained remission in a clinical setting at a very high risk of disease recurrence. For this reason, this maintenance is still continued following six maintenance courses delivered thus far.

## 5 Conclusions

The patient is presently in continuous hematological and molecular CR of his relapsed therapy-related MDS at more than 24 months since disease recurrence, which is quite satisfactory considering the limited chances of survival in patients with AML relapsing after allo-HSCT (2–5, 20–23). Based on both efficacy and tolerability, the maintenance program with sequential VEN and DLI courses has been continued at 3-month intervals for 2 years. We are planning to extend the maintenance at prolonged 4-month intervals, unless there are interruptions due to toxicity or disease reappearance.

The patient is a medical doctor. He is perfectly aware of his clinical situation, with previous occurrence of two different hematological malignancies. He is also aware of the innovative treatment employed for the management of his recent disease recurrence following allo-HSCT. Based on the excellent tolerability of the ongoing maintenance treatment along with the long-lasting hematological and molecular remission, he is willing to proceed with the maintenance therapy, possibly at the prolonged 4-month intervals. He has given a signed informed consent to have his clinical case reported to the scientific community.

Indeed, these are the key insights of the present clinical case report.

- There are novel treatment options for the management of MDS/AML relapse following allo-HSCT, with acceptable toxicity and reasonable efficacy even in high-risk conditions such as therapy-related MDS/AML.
- The combination of HMAs with anti-bcl-2 drugs and immunotherapy appears particularly suitable for post-graft MDS/AML recurrence.
- A maintenance therapy combining prolonged administration of anti-bcl-2 and immunotherapy courses appears feasible, and it should be further pursued for remission maintenance in high-risk patients following allo-HSCT.

## Data Availability

The datasets presented in this Case Report are not readily available because of serious privacy-related concerns. Requests to access the datasets should be directed to Corrado Tarella (corrado.tarella@unimi.it), pending a formal application and ethical approval.
